# A Case of Typical Carcinoid of the Larynx

**DOI:** 10.1155/2012/717251

**Published:** 2012-04-23

**Authors:** Shintaro Sato, Yuichiro Kuratomi, Fumio Yamasaki, Akira Inokuchi

**Affiliations:** ^1^Department of Otolaryngology-Head and Neck Surgery, Faculty of Medicine, Saga University, Saga 849-8501, Japan; ^2^Division of Pathology, Department of Pathology and Microbiology, Faculty of Medicine, Saga University, Saga 849-8501, Japan

## Abstract

We report herein a rare case of typical carcinoid occurring primarily in the epiglottis. The patient was a 70-year-old man. On initial examination, a polypoid lesion with irregular surface near the center right-hand side of the laryngeal surface of the epiglottis was observed, and a biopsy was performed. Pathological examination of the specimen suggested the possibility of adenocarcinoma. Surgical excision was performed by means of laryngomicrosurgery. A Weerda-type laryngoscope was used to open the larynx, supplemented by rigid nasal sinus surgery endoscopes, and the right-hand half of the epiglottis were excised was ensured using a CO_2_ laser. Postoperative pathological diagnosis was negative for adenocarcinoma and squamous cell cancer; typical carcinoid was diagnosed according to the World Health Organization criteria. Aspiration occurred postoperatively, swallowing training was therefore provided, and the patient was discharged from hospital 2 months after surgery when he was able to eat normally. As of 4 years after surgery, the patient remains under follow-up observation by means of PET-CT and neck, thoracic, and abdominal CT administered at appropriate intervals, but no findings indicating obvious recurrence or metastasis have been observed, and the patient displays good swallowing function.

## 1. Introduction

Neuroendocrine tumors of the larynx are rare. In 2003, however, the World Health Organization (WHO) issued a classification of laryngeal neuroendocrine tumors in line with that of lung tumors [1]. According to this new definition, typical carcinoid is regarded as a well-differentiated neuroendocrine carcinoma and is a rare tumor of the larynx [1–6]. We treated a patient with this rare typical carcinoid of the larynx by means of laryngomicrosurgery supplemented with rigid sinus surgery endoscopes and used a CO_2_ laser for *en bloc *resection and removal.

## 2. Case Study

**Patient**

A 70-year-old man.

**Main Complaint**

Tumorous mass of the epiglottis.

**Disease History**

When the patient underwent upper intestinal endoscopy at a local hospital in February 2008, a tumorous lesion was identified in the larynx. He was referred to his nearest ear, nose, and throat clinic, which found a tumor of the epiglottis and referred him to our department.

**Previous Medical History**

Coronary artery stent inserted after myocardial infarction at 65 years old.

**Smoking History**

Ten cigarettes/day from 20 to 65 years old; quit smoking thereafter.

**Drinking History**

Beer 350 mL/day (three *go* (a unit corresponding to 180 mL) of *shochu*/day up to 65 years old) were reported.

**Local Findings**

**Figure 1:**
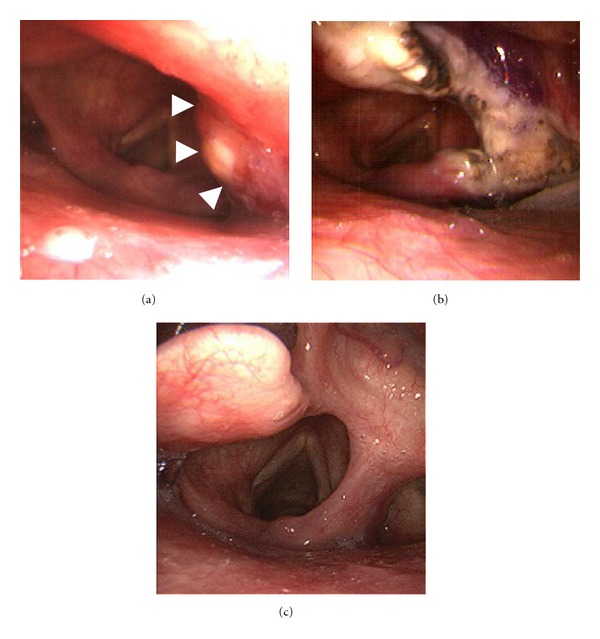
Laryngeal findings. (a) Before surgery, a somewhat uneven tumor in the submucosa can be seen on the right-hand side of the laryngeal surface of the epiglottis (arrow). (b) Four days after surgery. The right half of the epiglottis has been resected. The resection stump is covered with the white scab. (c) Two years after surgery. The wound has epithelialized well.

A bulky lesion with irregular surface was observed in the submucosa near the center right-hand side of the laryngeal surface of the epiglottis (Figure 1(a)). When a biopsy was taken from this lesion, pathological diagnosis showed the presence of carcinoma and the possibility of adenocarcinoma. T1N0M0 laryngeal cancer of the supraglottic type was diagnosed on the basis of this and other test findings.

**Disease Course**

The patient was scheduled for early hospital admission and treatment but developed subacute myocardial infarction the day after the initial examination in our department and was admitted by the Department of Cardiovascular Medicine of our hospital. Percutaneous coronary artery intervention was performed, and the patient returned to our department after a period of waiting for his overall condition to stabilize. No obvious changes in focal findings compared with those at initial examination 1 month earlier were seen at this time. As biopsy results had indicated the possibility of adenocarcinoma, radiotherapy was not expected to be very effective, and surgical resection was performed by laryngomicrosurgery using Shiotani's method.

**Figure 2:**
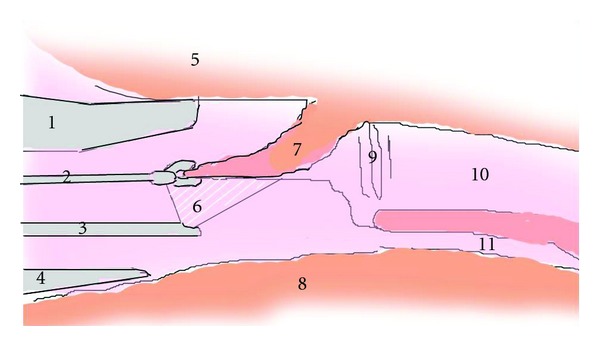
Surgical schema. The endotracheal intubation tube is omitted from this diagram. (1) Upper tip of Weerda-type laryngoscope. (2) Grasping forceps. (3) Rigid nasal sinus surgery endoscope at 70°. (4) Lower tip of Weerda type laryngoscope. (5) Lingual tonsil. (6) Scope of field of view of rigid endoscope. (7) Epiglottis. (8) Retropharyngeal wall. (9) Glottic space. (10) Trachea. (11) Esophagus.

**Surgery (Figure 2)**

A Weerda-type laryngoscope (KARL STORZ GmbH & Co. KG, Tuttlingen, Germany) was used to open the larynx. Rigid nasal sinus surgery endoscopes (30° and 70°) were inserted and retained from the side through the mouth by an assistant and used to supplement the field of view. The endoscopes ensured that the field of view of the laryngeal surface of the epiglottis was sufficient. The tip of the epiglottis was grasped with forceps, and the right-side half was excised and removed *en bloc* together with the tumor, while a safety margin was ensured using a CO_2_ laser. Figure 1(b) shows laryngeal findings 4 days after surgery.

**Histopathological Findings**

**Figure 3:**
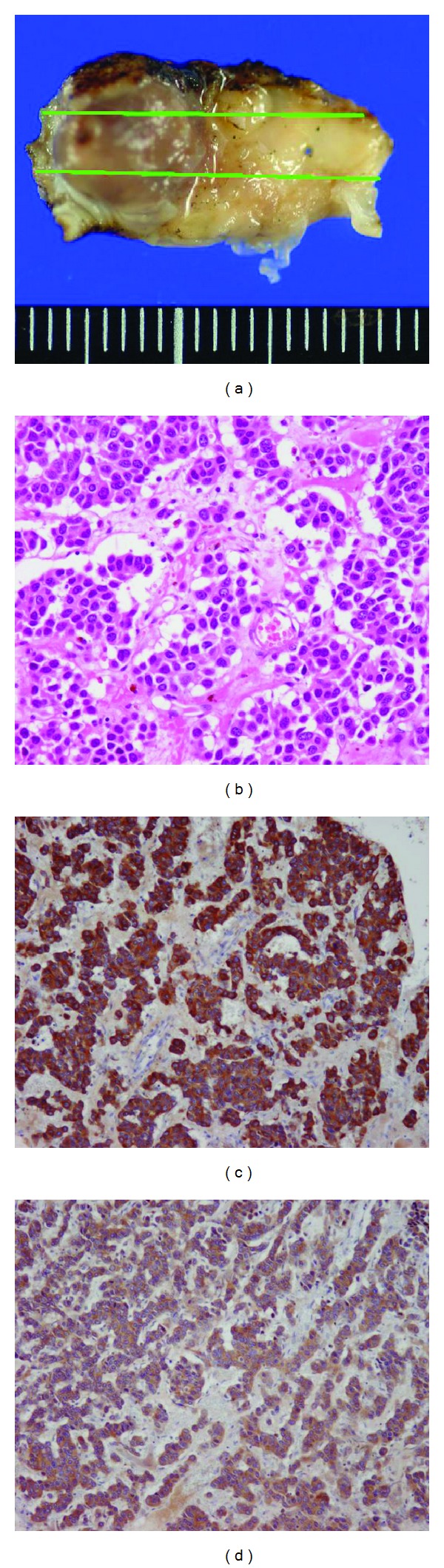
Histopathological findings. (a) Visual findings. An uneven tumor could be seen in the submucosa. Lines in the figure show the cross-sections used for specimen preparation. (b) Hematoxylin-eosin staining ×200. Tumor cells exhibit the so-called carcinoid pattern. Cell division was extremely low, at 1/10 hpf, and no vascular invasion or tendency to invade surrounding tissue was seen. (c) Chromogranin A ×100. Tumor cells were strongly positive. (d) Synaptophysin ×100. Tumor cells were strongly positive.

The resected specimen was fixed in 10% buffered formalin and processed for histology in the routine manner. Resected tissue showed an irregular surface tumor in the submucosa (Figure 3(a)). On hematoxylin and eosin staining (Figure 3(b)), tumor sections showed no clear lumen formation, intercellular bridges, or keratinization, ruling out adenocarcinoma and squamous cell cancer. Cell division was extremely low, at 1/10 HPF, and no vascular invasion or tendency to invade the surrounding tissue was evident. Immunohistochemical examination revealed that tumor cells were strongly positive for both chromogranin A (Figure 3(c)) and synaptophysin (Figure 3(d)). From these findings, the lesion was diagnosed as corresponding to a typical carcinoid in the WHO classification of neuroendocrine tumors of the larynx. The resection margin was also pathologically free of disease.

**Postoperative Course**

The prognosis for typical carcinoid is regarded as good compared with that for other types of neuroendocrine tumor, and as the resection margin was also sufficient, no postoperative treatment was administered. Aspiration occurred postoperatively and swallowing training was therefore provided, and the patient was discharged from hospital 2 months after surgery when he was able to eat normally. As of 4 years after surgery, the patient remains under follow-up observation using PET-CT and neck, thoracic, and abdominal CT administered at appropriate intervals, but no findings indicating obvious recurrence or metastasis have been observed, and the patient is able to swallow well. Current findings are shown in Figure 1(c).

## 3. Discussion

 Neuroendocrine tumors are comparatively rare in the head and neck region [1–6] and account for ≤1% of neoplasms occurring in the larynx [5]. The WHO has produced a classification of neuroendocrine tumors of the larynx based on its classification of neuroendocrine tumors of the lungs and elsewhere, and the 2003 version is shown in Table 1 [1]. We partly edited the table used by Ferlito et al., and a summary of neuroendocrine carcinoma is shown in Table 2 [5]. The ambitious review of neuroendocrine tumors of the larynx by Ferlito et al. describes a number of hypotheses that have been proposed regarding the histogenesis of neuroendocrine tumors of the larynx, but none of these can be described as a definitive theory [5, 6].

 Our patient showed a case of typical carcinoid (A in Table 1), corresponding to well-differentiated neuroendocrine carcinoma. Typical carcinoid is rare compared with the other two types of neuroendocrine carcinoma, and few reports have been described; malignancy as a neuroendocrine tumor is lower than that of the other two [1–6]. In terms of prognosis, Ferlito et al. point out that those cases reported as typical carcinoid may in fact have included some cases of atypical carcinoid, and the actual prognosis for typical carcinoid may be somewhat better [6]. However, despite occurring less often, metastases are still observed in 1/3 of cases [5], and continued followup of our case will be necessary in the future.

Conventional approaches such as laryngofissure offer a conceivable surgical procedure for tumors of the epiglottis, but Shiotani et al. recently reported partial laryngectomy by means of a video laryngoscope [7, 8]. Our hospital does not possess a video laryngoscope, but by combining rigid nasal sinus surgery endoscopes with a conventional laryngoscope, we were able to perform partial laryngectomy *en bloc* following the method of Shiotani et al.. Of course, resecting half of the epiglottis at once carries a risk of temporary dysphagia. In our case, however, swallowing training, carried out in collaboration between a doctor and a speech therapist, enabled the patient to eat normally, demonstrating that even potential postoperative dysphagia can be dealt with by the implementation of conscientious care.

 In summary, we treated a case of typical carcinoid occurring in the larynx. By combining rigid nasal sinus surgery endoscopes with a conventional laryngoscope, we were able to perform partial laryngectomy *en bloc*. The prognosis for typical carcinoid is considered to be better than that for atypical carcinoid or small-cell carcinoma, but as metastases are seen in one-third of cases, careful followup will be continued.

## Figures and Tables

**Table 1:** Classification of neuroendocrine tumors of the larynx by WHO, 2003.

Terminology	Synonyms
(A) Typical carcinoid	Carcinoid, well-differentiated (Grade I) neuroendocrine carcinoma
(B) Atypical carcinoid	Malignant carcinoid, moderately differentiated (Grade II) neuroendocrine carcinoma, large-cell neuroendocrine carcinoma^1^
(C) Small-cell carcinoma, neuroendocrine type^2^	Small-cell neuroendocrine carcinoma, poorly differentiated (Grade III) neuroendocrine carcinoma
(D) Combined small-cell carcinoma, neuroendocrine type, with non-small-cell carcinoma (squamous cell carcinoma, adenocarcinoma, etc.)	Combined small-cell carcinoma, composite small-cell carcinoma
(E) Paraganglioma	Nonchromaffin paraganglioma

^1^Some atypical carcinomas may fulfill diagnostic criteria for large-cell neuroendocrine carcinoma of the lung.

^2^Not all small-cell carcinomas of the larynx will show neuroendocrine differentiation.

**Table 2:** Comparison of laryngeal neuroendocrine carcinoma.

Features	Carcinoid tumor	Atypical carcinoid tumor	Small-cell neuroendocrine carcinoma
*Clinical *			
Age	Sixth decade	Sixth and seventh decades	Sixth and seventh decades
Sex ratio (M : F)	3 : 1	3 : 1	3 : 1
Location	Supraglottic	Supraglottic	Supraglottic
	submucosal	submucosal	ulcerated submucosal
Rate of metastases	33%	66.7%	90%
Paraneoplastic syndromes	Exceptional	Occasional	Occasional
Treatment	Surgery	Surgery	Systemic chemotherapy and irradiation
Prognosis	48.7% (∗)	36.5–48% (∗)	5–7.7% (∗)
		12.2–30% (∗∗)	
Reported cases	43	More than 350 cases	More than 180 cases

(∗) 5-year survival; (∗∗) l0-year survival.
